# High-Fidelity Imaging in Brain-Wide Structural Studies Using Light-Sheet Microscopy

**DOI:** 10.1523/ENEURO.0124-18.2018

**Published:** 2018-11-22

**Authors:** M. Caroline Müllenbroich, Ludovico Silvestri, Antonino P. Di Giovanna, Giacomo Mazzamuto, Irene Costantini, Leonardo Sacconi, Francesco S. Pavone

**Affiliations:** 1 National Institute of Optics, National Research Council, Sesto Fiorentino, 50019, Italy; 2 European Laboratory for Non-Linear Spectroscopy, LENS, Sesto Fiorentino, 50019, Italy; 3 University of Florence, Italy; 4 Department of Physics and Astronomy, University of Florence, Sesto Fiorentino 50019, Italy

**Keywords:** light-sheet microscopy, structural imaging, whole-brain imaging, mouse, vascular/neuronal networks

## Abstract

Light-sheet microscopy (LSM) has proven a useful tool in neuroscience to image whole brains with high frame rates at cellular resolution and, in combination with tissue clearing methods, is often employed to reconstruct the cyto-architecture over the intact mouse brain. Inherently to LSM, however, residual opaque objects, always present to some extent even in extremely well optically cleared samples, cause stripe artifacts, which, in the best case, severely affect image homogeneity and, in the worst case, completely obscure features of interest. Here, demonstrating two example applications in intact optically cleared mouse brains, we report how Bessel beams reduce streaking artifacts and produce high-fidelity structural data for the brain-wide morphology of neuronal and vascular networks. We found that a third of the imaged volume of the brain was affected by strong striated image intensity inhomogeneity and, furthermore, a significant amount of information content lost with Gaussian illumination was accessible when interrogated with Bessel beams. In conclusion, Bessel beams produce high-fidelity structural data of improved image homogeneity and might significantly relax demands placed on the automated tools to count, trace, or segment fluorescent features of interest.

## Significance Statement

The standard way to measure neuronal structure in light-sheet microscopy (LSM) applies Gaussian beams, a method prone to artifacts introduced by residual opaque objects present even in extremely well optically cleared samples. These objects cause streaky shadows, which severely affect image homogeneity and, furthermore, obscure features of interest. In measurements using Bessel beam illumination; however, high-fidelity imaging of micro-anatomic detail is restored. Since whole mouse brain datasets now routinely comprise several terabytes, automated tools to count, trace, or segment features of interest are needed to extract meaningful insights. This is why high-fidelity structural imaging has the potential to relax computational demands on the algorithms used to turn data into knowledge.

## Introduction

The brain is an immensely complex entity in which structure and function are intricately correlated and best elucidated on an organ-wide scale at cellular resolution. One technique, which is particularly suited for whole-brain investigations, is light-sheet microscopy (LSM; Siedentopf and Zsigmondy, 1902) due to its intrinsic optical sectioning capabilities, fast acquisition rates, and low photobleaching. In LSM, fluorescence is excited in a thin sheet of excitation light that coincides with the focal plane of a perpendicularly placed detection objective (Huisken et al., 2004). By combining advanced tissue clearing methods (Richardson and Lichtman, 2015; Silvestri et al., 2016; Tainaka et al., 2016) with LSM, neuronal and vascular cyto-architecture can be reconstructed over cm-sized samples like the entire mouse brain (Dodt et al., 2007; Susaki et al., 2014; Pan et al., 2016) for quantitative structural studies.

Despite its intrinsic advantages, the very nature of uncoupled, perpendicular optical pathways for fluorescence excitation and detection entails a different set of drawbacks unique to LSM. Previously published studies (Chen et al., 2016) have shown that refractive heterogeneities, always present to some extent even in extremely well optically cleared samples, lead to a loss of spatial resolution and a concomitant degradation in sensitivity and contrast. Particular to LSM are dark shadows that appear whenever the fluorescence-exciting light sheet is interrupted by scattering or absorbing obstacles in the form of bubbles, dust or other refractive index mismatches. At best, these dark shadows severely affect image homogeneity; at worst, they completely obscure any feature of interest in their path. Considering the increasingly large dataset sizes routinely produced in high-throughput LSM, high demands are placed on the automated tools to count, trace, or segment the fluorescent features of interest. Consequently, background uniformity and indeed high-fidelity imaging are paramount to facilitate the extraction of meaningful insights from terabytes of data, such as cell counts or segmentation of axons or blood vessels.

Previously published work to alleviate the problem of shadowing on the microscope system side includes pivoting the light sheet rapidly over a few degrees (Huisken and Stainier, 2007), a method incompatible with confocal line detection (Baumgart and Kubitscheck, 2012). Employing double-sided illumination (Dodt et al., 2007), a necessity when imaging intact mouse brains due to their sheer size, is not capable to improve striping since the distal light sheet cannot be made to coincide with the proximal one after having passed through the majority of the sample. Other published attempts to tackle streaky artifacts are based on post-processing, for example, in the frequency domain using nonsubsampled contourlet transform methods (Liang et al., 2016). Although successfully demonstrated, this and other algorithms are often computationally intensive and therefore neither compatible with *in vivo* imaging where the correction is needed at run time nor with structural imaging where terabyte-sized datasets are routinely produced in automated high-throughput microscopes.

Aiming for an optical solution to streaking artifacts, here, we apply Bessel beams (Durnin et al., 1987) to LSM to investigate biological samples for the high-fidelity interrogation of their structure. Bessel beams have been widely studied in various optical imaging methods as their propagation invariant, diffraction-free properties make them highly attractive, for example, to extend the useful field of view or depth of field (Lorenser et al., 2014; Lu et al., 2017). The “self-healing” properties of Bessel beams, on the other hand, properties enabled by the energy stored in the concentric rings replenishing the central lobe if it encounters a partial obstruction (Bouchal et al., 1998; Garcés-Chávez et al., 2002), make them robust to imaging in scattering media (Fahrbach et al., 2010). A penalty has to be paid, however, for the self-healing capabilities of Bessel beams. The on-axis irradiance in Bessel beams is lower compared to peak Gaussian beams of the same power due to the energy stored in the outer rings of the Bessel beam. Additionally the outer rings can generate out-of-focus fluorescence which means that, in general, images acquired with a Bessel beam exhibit lower contrast and inferior optical sectioning compared to Gaussian illumination, though confocal line detection (Fahrbach and Rohrbach, 2012) can be applied to ameliorate signal to background. A Bessel beam’s central core can be extremely narrow without being subject to diffraction (McGloin and Dholakia, 2005), leading to the application of Bessel beams to LSM geared toward isotropic resolution (Planchon et al., 2011; Gao et al., 2014) or studies of the scattering properties of the sample and the self-reconstructing properties of the Bessel beam itself (Fahrbach et al., 2010, 2013a). Finally, two groups have published technological development concerning Bessel beams applied to LSM (Zhang et al., 2014; Zhao et al., 2016).

Here, we report on the application of Bessel beams in LSM to investigate more specific biological samples in two typical applications, structural imaging of neurons and vasculature in the whole mouse brain. Using a direct comparative analysis between Bessel and Gaussian illumination, we provide supporting evidence and quantification of artifacts introduced by Gaussian illumination and further demonstrate that Bessel beams provide superior image homogeneity and indeed reveal structural information lost when applying standard illumination.

## Materials and Methods

### Animals

Transgenic mouse strains showing distinct fluorescent labeling where used for the experiments shown: Thy1-GFP-M with a sparse labeling across the entire encephalon Feng et al., 2000 (*N* = 2); B6N.Cg-*Sst^tm2.1(cre)Zjh^/J ×* B6.Cg-*Gt(ROSA)26Sor^tm9(CAG-tdTomato)Hze^/J* (Sst-tdTomato) labeling all somatostatin-positive neurons (*N* = 3); *Vip^tm1(cre)Zjh^/J ×* B6.Cg-*Gt(ROSA)26Sor^tm9(CAG-tdTomato)Hze^/J* (VIP-tdTomato) labeling all vasoactive intestinal peptide-positive neurons (*N* = 4); B6.Cg-*Pvalb^tm1.1(cre)Aibs^ ×* B6.Cg- *Gt(ROSA)26Sor^tm9(CAG-tdTomato)Hze^* (PV-tdTomato) labeling all parvalbuminergic neurons Madisen et al., 2010 (*N* = 1); B6.129(Cg)- *Fos^tm1.1(cre/ERT2)Luo^/J ×* B6.Cg-*Gt(ROSA)26Sor^tm9(CAG-tdTomato)Hze^* (FosTRAP) labeling all neurons expressing the immediate early gene c-Fos (Guenthner et al., 2013; *N* = 2). In this latter model, fluorescence expression has been induced by intraperitoneal injection of tamoxifen (as described in detail in Guenthner et al., 2013) in home cage conditions. All mice originate from the Jackson Labs and animals where raised and caged according to the Italian law, under authorization n. 790/2016-PR by the Italian Ministry of Health.

### Perfusion protocol

Brain vasculature labeling of a male adult Thy1-GFP-M mouse (*N* = 1) was performed using the staining protocol described in (Tsai et al., 2009), but replacing fluorescein-albumin with tetramethylrhodamine-albumin to avoid spectral overlap with the GFP expressed by neurons. After deep anesthesia with isoflurane inhalation, the mouse was transcardially perfused with 20–30 ml of 0.01 M of PBS solution (pH 7.6) and then with 60 ml of 4% (w/v) paraformaldehyde (PFA) in PBS. This was followed by perfusion with 10 ml of a fluorescent gel perfusate, with the body of the mouse tilted by 30°, head down, to ensure that the large surface vessels remained filled with the gel. The body of the mouse was submerged in ice water, with the heart clamped, to rapidly cool and solidify the gel as the final portion of the gel perfusate was pushed through. The brain was carefully extracted to avoid damage to pial vessels after 30 min of cooling and incubated overnight in 4% PFA in PBS at 4°C. The day after the brain was rinsed three times in PBS. All experimental protocols were designed in accordance with Italian laws and were approved by the Italian Minister of Health (authorization 790/2016-PR).

### Gel preparation

The gel was prepared as described previously (Tsai et al., 2009) but replacing fluorescein-albumin with tetramethylrhodamine-albumin (A23016, Thermo Fisher Scientific). First, a 2% (w/v) of porcine skin gelatin type A (G1890; Sigma) was prepared in boiling PBS and allowed to cool to below 50°C. The gelatin was then combined with 0.05% (w/v) of tetramethylrhodamine-albumin (A.P. Di Giovanna et al., 2018). The solution was maintained at 40°C while stirring before the perfusion.

### Photothrombotic stroke protocol

The surgical procedure was performed under zoletil (50 mg/kg) and xylazine (9 mg/kg) anesthesia. The skin over the skull was cut and the periostium was removed with a blade. 5 min after i.p. injection of rose bengal (10 mg/ml), white light from an LED lamp (CL 6000 LED, Zeiss) was focused on the primary motor cortex with a 20× objective (EC Plan Neofluar NA 0.5, Zeiss) and illuminated for 15 min (Labat-gest and Tomasi, 2013). Afterward, the skin over the skull was sutured and the animal (*N* = 1) was placed in a recovery cage until full recovery.

### Whole-brain clearing procedure

Mouse brains of male adults (*N* = 12) were cleared using the CLARITY technique (Chung et al., 2013), applying the passive clearing procedure. Fixed mouse brains were incubated in hydrogel solution [4% (wt/vol) acrylamide, 0.05% (wt/vol) bis-acrylamide, 0.25% (wt/vol) VA044] in 0.01 M PBS at 4°C for one week. Samples were degassed and incubated at 37°C for 3 h to allow hydrogel polymerization. Subsequently, the brains were extracted from the polymerized gel and incubated in clearing solution [200 mM sodium borate buffer, 4% (wt/vol) sodium dodecyl sulfate (pH 8.5) at 37°C for one month while gently shaking]. The samples were then washed with PBST (0.1% Triton X-100 in 1× PBS) twice for 24 h each at room temperature. CLARITY-processed mouse brains were optically cleared using 2,2’-thiodiethanol (TDE), as described in (Costantini et al., 2015). After PBST washing, the brains were serially incubated in 50 ml of 30% and 63% (v/v) TDE in 0.01 M PBS (TDE/PBS), each for 1 d at 37°C while gently shaking. After TDE clearing the brains were ready for imaging.

### Optical characterization of the microscope

To measure the point spread function (PSF) of the microscope, fluorescent beads of 100 nm diameter (FluoSpheres F8803, ThermoFisher Scientific) were imaged in a z-stack. Several tens of beads were automatically extracted and distilled into a PSF using commercial software (PSF distiller, Huygens Software, Scientific Volume Imaging BV). The beam waist, Rayleigh range and beam numerical aperture of the Gaussian beam were extracted by fitting Gaussian profiles over the stationary excitation beam, plotting the respective full width at half maximum (FWHM) against the axial distance of the beam and fitting the resulting curve to a hyperbolic function.

### Image stitching and analysis

The LSM for structural imaging produces a series of 3D stacks with regions of superimpositions. To achieve a 3D image of whole specimens we used ZetaStitcher, a dedicated stitching tool that was developed in house and is freely downloadable (ZetaStitcher, 2017). Graphs were done with OriginPro 9.0 (OriginLab Corporation) and data analysis with MATLAB (MathWorks). For neuronal imaging, stitched images encompassing the entire mouse brains were analyzed in a custom-written MATLAB program that used spatial filtering in the Fourier domain to obtain stripe-free images in the two perpendicular orientations. After inverse fast Fourier transform, the resulting images were subtracted from the original image to obtain the images of the stripes parallel and perpendicularly to the light-sheet illumination direction which were then binarized and expressed as a percentage of the area of the brain. For vasculature imaging, image stacks were analyzed using both Fiji (Schindelin et al., 2012) and Amira 5.3 (Visage Imaging) software. 3D renderings of stitched images were produced from downsampled files using the Amira Voltex function. The Filament Editor of Amira was used to manually trace vessels segments. Automatic segmentation of vasculature stacks was performed with Fiji by first aligning and registering both stacks to each other using the rigid body modality in the StackReg plugin. A γ of 1.1 was applied to both stacks over the entire image to enhance dimmer structures and an auto threshold using IsoData was applied. The Manders coefficients were calculated using the JACoP plugin (Bolte and Cordelières, 2006).

### Statistical analysis

To estimate the amount of striping present in whole-brain acquisitions using Gaussian illumination, *N* = 10 animals were used. For the remaining structural imaging experiments, no sample size was computed since we investigated artifacts that are intrinsic to the microscopy method used (static shadow artifacts in LSM). Additionally, since the measurements are indeed whole mouse brain acquisitions, even with a sample size of *N* = 1 mouse, millions of images and terabytes of data were generated, which was more than enough to study the effects of streaking artifacts. As such, the results were illustrated on one dataset for each case study, that is: *N* = 1 for neuronal imaging and *N* = 1 for vasculature imaging. Each whole-brain tomography was repeated twice in immediate succession. Once using Gaussian illumination and then again using Bessel beam illumination. There is no biological replication since only *N* = 1 mouse brain was used for each comparison. There is no technical replication since each tomography was only made once. Outliers do not apply to this case/are non-existent.

### Gaussian and Bessel beams

**Figure 1.**
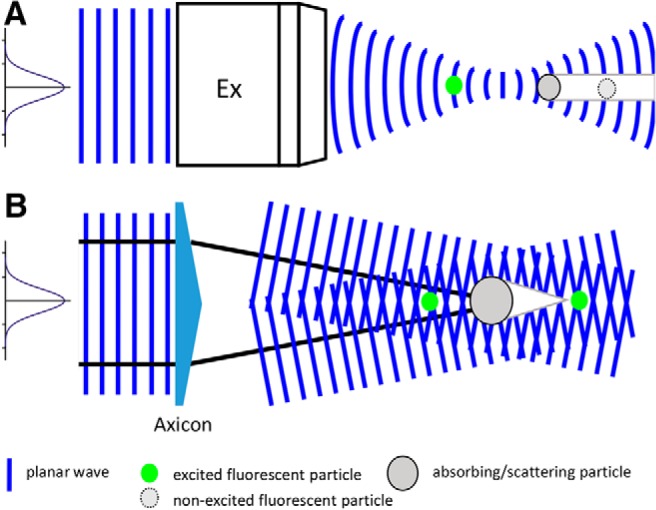
Shadowing with Gaussian and Bessel beams. ***A***, Generation of shadows when focusing a Gaussian beam with a microscope objective (Ex). Fluorescent particles positioned in the elongated shadow cannot be excited. ***B***, For Bessel beams, here generated by a Gaussian beam impinging on a conical lens called axicon, the optical power stored in the concentric rings can regenerate the initial beam profile in the reconstruction region behind the shadow zone. Fluorescent particles behind the conical shadow can be excited and therefore imaged.

The typical shape for laser radiation propagating in the free space is the Gaussian beam, which takes the name from the shape of the transverse intensity profile. The Gaussian beam is composed by the superposition of plane waves with different inclinations, with an angular distribution (the so-called “angular spectrum”; Goodman, 2005) peaked on the propagation axis, with (again) a Gaussian distribution (Goodman, 2005). Since most of the energy is carried by the waves propagating along the axis direction, the presence of any scattering or absorbing particle along the beam results in a dark shadow (Fig. 1*A*).

Bessel beams are instead the result of the linear superposition of plane waves arranged on the surface of a cone (Fig. 1*B*), giving rise to a transverse intensity profile in the form of a Bessel function (Durnin et al., 1987). The peculiar angular spectrum of these beams confers their well-known self-healing property (Bouchal et al., 1998), as scattering or absorbing particles project only a conical shadow, and afterward the beam propagates unhindered (Fig. 1*B*). A simple and economically convenient way to generate Bessel beams is through the use of a conical lens called axicon (Indebetouw, 1989).

**Figure 2.**
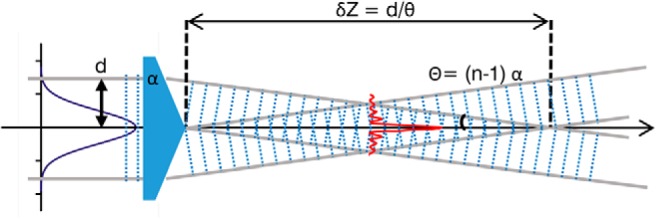
Generation of a Bessel beam using an axicon lens. The characteristic transverse *J*_0_ Bessel beam profile of a central lobe with concentric rings is created within the propagation length *δ Z* of the axicon which is a function of the input beam radius *d* and the angle *α* of the conical lens.

The Bessel beam parameters are related to the diameter of the incoming Gaussian beam and to the axicon aperture through a series of simple equations 1, 2, 3. The depth of focus *δ Z* (Fig. 2) is given by:

(1) $$\delta Z=\;\frac{d}{\Theta }$$

where *d* is the radius of the incoming Gaussian beam and Θ is aperture of the cone of light that is creating the Bessel beam.

This angle is related to the refractive index *n* and the aperture angle of the conical lens *α* by:

(2) Θ=(n−1)α

The radius of the central core of the Bessel beam is given by:

(3) $$r_{c}=\frac{2.405\lambda }{2\pi \sin\theta }$$

where *λ* is the wavelength of the excitation light.

**Figure 3.**
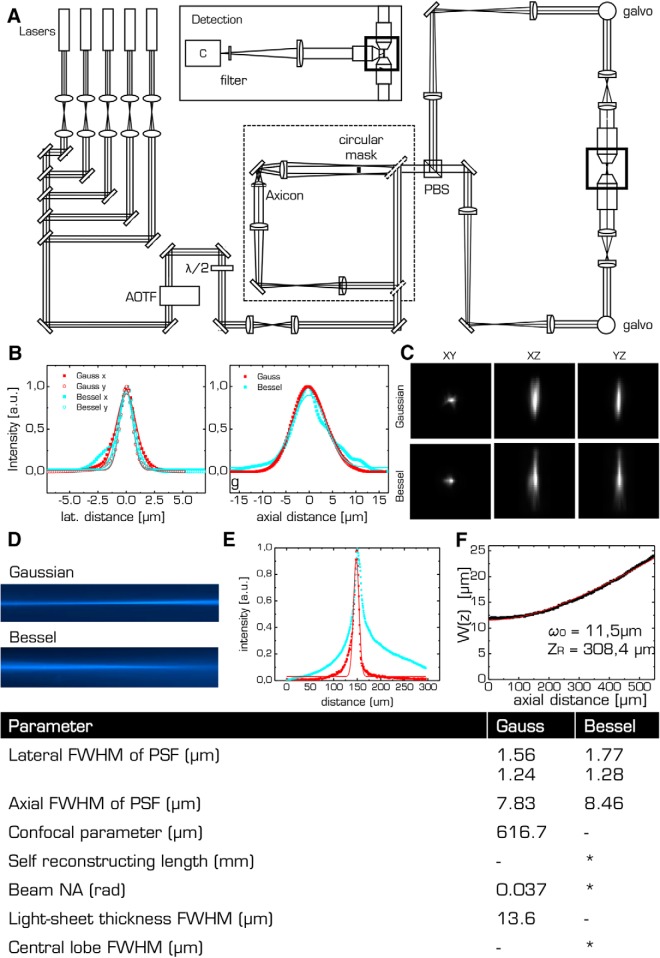
***A***, The custom-made LSM uses double sided illumination by either Gaussian or Bessel beams. ***B***, Measured PSFs for the lateral and axial direction for Gaussian (red) and Bessel illumination (cyan) using fluorescent beads. The FWHMs of the PSF are reported in the table for Gaussian and Bessel illumination, respectively. ***C***, PSFs for Gaussian and Bessel illumination. ***D***, Longitudinal beam profile. ***E***, Transversal profile for the Gaussian (red) and Bessel beam (cyan). ***F***, Beam width *ω*(*z*) of the Gaussian beam extracted from profile shown in ***F***. Red line indicates fit to hyperbolic function. The beam waist *ω*_0_, the Rayleigh range *Z_R_*, and the beam NA were extracted from the fit. Bottom, Table of all beam parameters. * indicates theoretically derived values.

A quick inspection of Equations 1, 3 readily shows another distinct feature of Bessel beams, i.e., the independence of the depth of focus and of the core radius. Indeed, *d* and *α* can be properly engineered to obtain beams with tiny central core that propagate for very long distances. This is in contrast with the conventional Gaussian beams, where the depth of focus and the beam waist are always directly proportional (Saleh et al., 1991). Bessel beams therefore allow the creation of thin and long sheets of light in LSM (Fahrbach et al., 2010). On the downside, the outer rings of the Bessel beam introduce out-of-focus excitation (see projection of a Bessel beam in Fig. 3*E*) that reduces the optical sectioning capabilities of the microscope. However, this problem can be slightly mitigated by means of confocal line detection (Fahrbach and Rohrbach, 2012) or more significantly by the use of sectioned Bessel beams (Fahrbach et al., 2013b).

## Results

### Custom LSM for structural studies of whole optically cleared mouse brains

A custom-made LSM, specifically designed for the imaging of whole optically cleared mouse brains (Müllenbroich et al., 2015), was functionally expanded to incorporate Bessel beam illumination (Fig. 3, dashed box) and used for all structural imaging experiments. The microscope was equipped with an alternative excitation light path using two flip mirrors that directed the light toward an axicon (AX252-A, *α* = 2°, Thorlabs) which created the Bessel beam by superimposing plane waves whose wave vectors lie on a cone (Indebetouw, 1989). The Bessel beam was filtered in a Fourier plane with a circular spatial filter to eliminate any residual Gaussian contribution. The Gaussian or Bessel beam, respectively, was split by a polarizing beam splitter and subsequently scanned by two galvo mirrors (6220H, Cambridge Technology) to create a light sheet that illuminated each brain half from its respective side. The excitation objectives (Plan Fluor EPI, 10×, 0.3NA, WD 17.5 mm, Nikon), covered with a protective coverslip, projected the light sheet into the focal plane of the perpendicularly placed detection objective (XLPLN10XSVMP, 10×, 0.6NA, WD 8 mm, Olympus, see inset of Fig. 3*A* Detection) specifically designed for immersion in high-refractive index media and featured a correction collar for the refractive index of the immersion solution, ranging from 1.33 to 1.52. A tube lens formed an image onto the sensor of a fast sCMOS camera (Orca Flash4.0 v2.0, Hamamatsu) whose line-by-line readout was synchronized to each step of the galvo mirrors to achieve confocal line detection. Appropriate bandpass filters were used to reject excitation light. For vasculature imaging, excitation was *λ* = 561 nm and an acousto-optic tunable filter (AOTFnC- 400.650-TN, AA Opto-Electronic) was used to regulate laser power. The samples were place in a quartz cuvette (3/Q/15/TW, Starna Scientific) containing the immersion medium (*n* = 1.45, 63% TDE in PBS) and placed in a custom-made chamber filled with the same immersion medium. The samples were mounted on a high-accuracy, motorized x-, y-, z-, Θ-stage (M-122.2DD and M-116.DG, Physik Instrumente) which allowed free 3D motion and rotation. The microscope was controlled via custom software written in LabVIEW 2012 (National Instruments) using the Murmex library (Distrio, Amsterdam, The Netherlands).

The optical performance of the microscope was quantified by determining the FWHM in the radial and axial directions (Fig. 3*B*) of the point-spread function for Gaussian and Bessel beam modality, respectively (Fig. 3*C*). The lateral and axial FWHMs were 1.56 and 7.83 µm for the Gaussian beam, respectively, whereas those values where only slightly larger for Bessel beam illumination (1.77 and 8.47 µm, respectively). In LSM, lateral resolution is a standard Airy function determined solely by the wavelength and the NA of the detection objective, which explains the good agreement in lateral resolution between Gaussian and Bessel beam illumination.

The longitudinal excitation beam profiles for Gauss and Bessel are shown in Figure 3*D* and their transversal projections, extracted from the center of those images, are presented in Figure 3*E*. These projections along the detection axis, not to be confused with beam cross sections, explain the similar axial resolution between Gaussian and Bessel beam illumination considering that confocal line detection is employed in the setup.

For the Gaussian beam, a confocal parameter *b* of 1.06 mm and a light-sheet thickness of 15 mm (FWHM) where extracted from the longitudinal profile. For Bessel beam illumination, the theoretically calculated self-reconstruction length was 1.7 mm, whereas the FWHM of the central lobe was determined at ∼1.2 µm; however, the actual thickness of the light sheet, capable of exciting fluorescence, is considerably larger taking the Bessel beam side lobes into account. Indeed, Bessel beams provide higher sectioning capabilities only when coupled with structured illumination or non-linear excitation (Planchon et al., 2011). All optical values are summarized in the table at the bottom of Figure 3.

### Streaking artifacts severely affect image homogeneity

Here, we present the effects of streaking artifacts in structural imaging aimed at obtaining cellular-resolution maps of the anatomy over the intact optically cleared mouse brain in two case studies, firstly targeting neurons and secondly, targeting vasculature. It is apparent that in all images obtained using Gaussian illumination image homogeneity is strongly affected and dark shadows obscure microscopic anatomic features of interest. Notably those same identical features remain clearly visible when using Bessel beam illumination.

**Figure 4.**
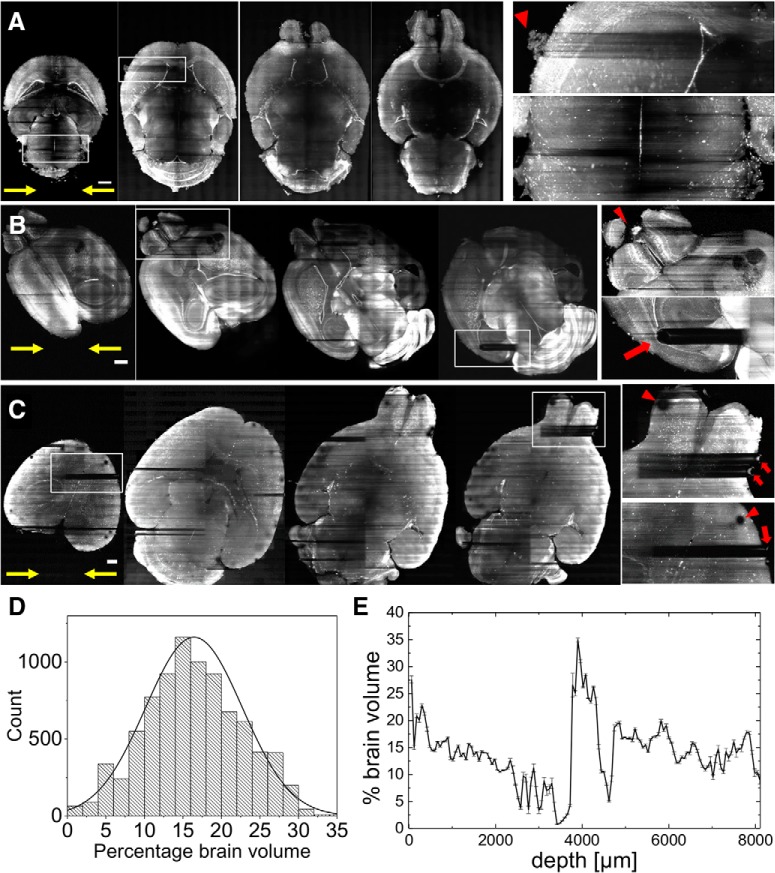
Striping with Gaussian illumination and estimation of the volume affected by severe streaking parallel to the illumination direction (yellow arrows). ***A***, Axial sections through the brain of a FosTRAP mouse. Scale bar: 1 mm. Inset, top, Striping caused by absorbing structures on the surface (red arrowhead). Bottom, Striping caused toward the center line of the brain by progressive absorption. ***B***, Axial sections through the brain of a PV-tdTomato mouse. Scale bar: 1 mm. Inset, top, Striping caused by a absorption of a particularly bright part of the olfactory bulb (red arrowhead). Bottom, Shadow caused by an internal structure, presumable due to incomplete optical clarification (red arrow). ***C***, Axial sections through the brain of a FosTRAP mouse. Scale bar: 1 mm. Inset, Striping caused by bubbles settling on the brain surface (red arrows). The same bubbles cause the circular shadows on the detection path (red arrow heads). ***D***, Percentage of the brain volume affected by streaking in *n* = 8446 stitched whole-brain images from *N* = 10 animals. Line is fit to Gaussian. ***E***, Variation of striping within axial sections throughout the depth of one mouse brain (shown in ***C***).

Figure 4 summarizes shadowing artifacts caused by Gaussian illumination in neuronal imaging demonstrated on axial sections of intact mouse brains (Fig. 4*A–C*) using a custom-made LSM (Fig. 3). Note, that each half of the intact brain is illuminated from its respective side (yellow arrows). The insets illustrate various causes of striping artefacts, including absorption by bright structures, refraction by bubbles on the brain surface, progressive attenuation of the excitation light and incomplete clearing. Substantial dark horizontal shadows traverse each brain half and completely obscure any features in their path. Using spatial filtering in Fourier space, an estimate of striping was obtained in the parallel and perpendicular (control) direction with regard to illumination. Using whole-brain acquisitions from *n* = 8446 stitched slices in *N* = 10 animals, 16.4 ± 6.3% of the entire brain volume were obscured by stripes (peak of Gaussian fit, error is SD). This represents a conservative estimate since images used for analysis were downsampled to a pixel size of 10.4 µm to reduce computing time. To illustrate a typical variation within one single brain, Figure 4*E* shows the evolution of striping in axial whole-brain sections with depth throughout the intact brain.

**Figure 5.**
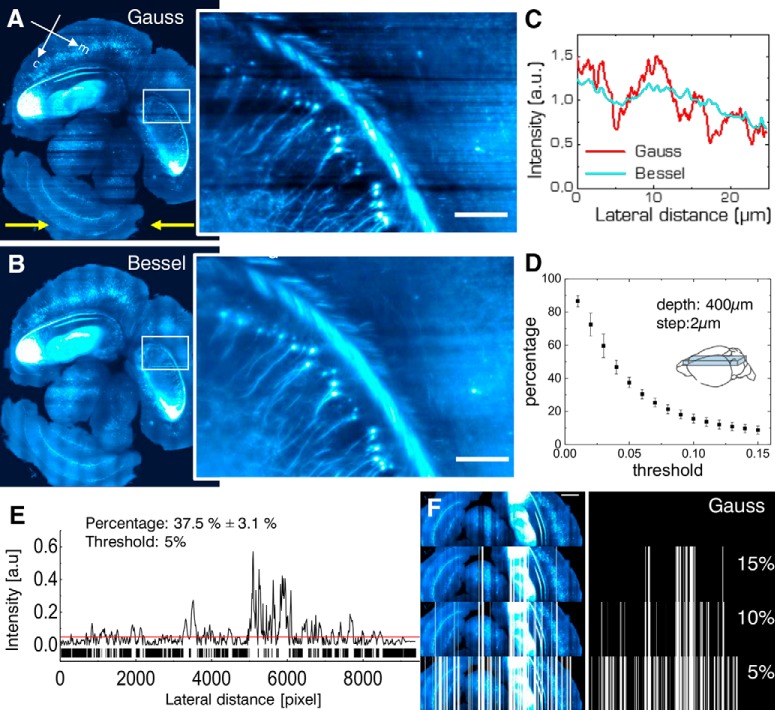
Structural neuron imaging. ***A***, Maximum intensity projection over 20 µm of a Thy1-GFP-M axial mouse brain section imaged with a Gaussian and a Bessel beam (***B***). Yellow arrows indicate direction of light-sheet propagation. Each half of the brain was excited by one light sheet, respectively. White box marks position of details in the hippocampus affected by streaking artifacts for Gaussian and Bessel beam illumination, respectively (insets). Scale bar: 10 µm. ***C***, Line profile averaged over the entire height of the inset evidences the shadows as drops in the red curve. ***D***, Sensitivity of striping to chosen threshold. ***E***, Calculating the absolute value of the difference in intensity line profile between Gauss and Bessel allows to estimate the area affected by streaking artifacts by applying a threshold (here 5%). Applying this threshold to stitched images of half a brain (***F***, bottom row) over a depth of 400 µm with a step size of 2 µm yielded that 37.5 ± 3.1% (error is SD) of the dataset was affected by streaking artifacts.

To analyze striping in more detail, we next looked at an axial slab encompassing the entire brain of an intact Thy1-GFP-M mouse over a depth of 400 µm (stitched from image stacks with step size 2 µm). A maximum intensity projection of 20 µm imaged with Gaussian illumination (Fig. 5*A*) evidences again dark horizontal shadows which are further illustrated in an inset detailing the hippocampus. The yellow arrows mark the bidirectional illumination. By contrast, the same area acquired with Bessel beam illumination (Fig. 5*B*, inset) shows improved image homogeneity and the absence of strong shadowing. To quantify the extent of the area affected by strong image inhomogeneity, we calculated the line profiles obtained over the entire height of the image for Gauss and Bessel illumination, respectively (Fig. 5*C*) and further binarized their absolute difference (Fig. 4*E*) with respect to a user-selected threshold to obtain a pattern similar to a bar code. By superimposing this bar pattern to the original image (Fig. 5*F*) the percentage of 2D area affected by streaking inhomogeneity was estimated. Averaged over the slab and using a threshold 5%), we calculated that 37.5 ± 3.1% (error is SD) of the images were affected by streaking. The percentage of brain area affected by streaking as a function of the chosen threshold is presented in Figure 5*D*.

### Streaking artifacts obscure microscopic features of interest

**Figure 6.**
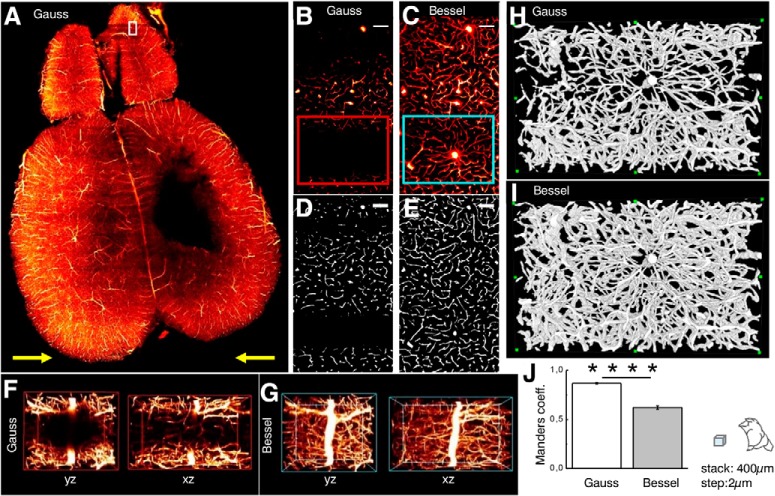
Vascular structural imaging. ***A***, Adult Thy1-GFP-M mouse with photothrombotic stroke in the primary motor cortex and brain vasculature labeling with tetramethylrhodamine-albumin imaged with Gaussian illumination. Yellow arrows indicate light-sheet propagation. White box in the olfactory bulb corresponds to images ***B–E***. Strong shadows obscure even large vessels when illuminated with Gaussian (***B***) but not with Bessel beam illumination (***C***). Scale bar: 10 µm. Automated segmentation based on simple thresholding for Gaussian (***D***) and Bessel beam illumination (***E***). ***F***, ***G***, Isometric views of red (cyan) box in ***B***, ***C*** shown along yz and xz for Gaussian (Bessel) illumination. ***H***, ***I***, 3D projections of the segmented data corresponding to the red and cyan ROIs indicated in ***B***, ***C***. ***J***, Manders coefficients averaged over a 400-µm stack comprising images in ***D***, ***E*** for Gaussian and Bessel illumination. The fraction of total intensity in the Bessel channel located in pixels of non-zero intensity in the Gaussian channel was 0.62 *±* 0.02, whereas the corresponding value for the Gaussian channel was 0.87 *±* 0.01 (*p <* 0.0001, paired *t* test, *n* = 39, error is SEM). See also Movies 1–8 for this figure. ****p ≤ 0.0001.

The effects of streaking artifacts on vasculature imaging are summarized in Figure 6 where an axial section of mouse brain vasculature illuminated with a Gaussian beam is shown (Fig. 6*A*). The white box in the olfactory bulb marks the position of details shown in Figure 6*B*,*C* for Gaussian and Bessel illumination, respectively (Movies 1 and 2). The red box marks the position corresponding to the isometric view along yz and xz illustrated in Figure 6*F* for Gaussian illumination whereas the cyan box is depicted in isometric view using Bessel beam illumination in Figure 6*G*. Although the whole-brain dataset appears to be of high quality, strong shadows in the Gaussian case completely obscure even large vessels that remain visible when illuminated with a Bessel beam (Movies 5 and 6). Using an automated segmentation based on simple thresholding (Fig. 6*D*,*E*; Movies 3 and 4; Fig. 6*H*,*I*, 3*D* projections; Movies 7 and 8), the Manders coefficients were averaged throughout the stack and are reported in Figure 6*J*. Note, that values range from 0 to 1 and express the fraction of intensity in the Gaussian channel that is located in pixels where there is non-zero intensity in the Bessel channel and vice versa. Throughout the depth of the stack the fraction of total intensity in the Bessel channel located in pixels of non-zero intensity in the Gaussian channel was 0.62 *±* 0.02, whereas the corresponding value for the Gaussian channel was 0.87 *±* 0.01 (*p <* 0.0001, paired *t* test, *n* = 39, error is SEM). Broadly speaking, this signifies that while 87% of the image content present in the Gaussian channel was also present in the Bessel channel only 62% of the image content present in the Bessel channel had corresponding content in the Gaussian channel.

**Movie 1.** Raw data of the vasculature of a Thy1-GFP-M mouse labelled with tetramethylrhodamine-albumin imaged with Gaussian illumination. See Figure 6*B* for details.

**Movie 2.** Raw data of the vasculature of a Thy1-GFP-M mouse labelled with tetramethylrhodamine-albumin imaged with Bessel beam illumination. See Figure 6*C* for details.

**Movie 3.** Segmented data of the vasculature of a Thy1-GFP-M mouse labelled with tetramethylrhodamine-albumin imaged with Gaussian illumination. Automated segmentation was based on simple thresholding. See Figure 6*D* for details.

**Movie 4.** GFP-M mouse labelled with tetramethylrhodamine-albumin imaged with Bessel beam illumination. Automated segmentation was based on simple thresholding. See Figure 6*E* for details.

**Movie 5.** 3D projection of raw data of the vasculature of a Thy1-GFP-M mouse labelled with tetramethylrhodaminealbumin imaged with Gaussian beam illumination. A look-up table was applied for clarity. See Figure 6*F* for details.

**Movie 6.** 3D projection of raw data of the vasculature of a Thy1-GFP-M mouse labelled with tetramethylrhodaminealbumin imaged with Bessel beam illumination. A look-up table was applied for clarity. See Figure 6*G* for details.

**Movie 7.** 3D projection of segmented data of the vasculature of a Thy1-GFP-M mouse labelled with tetramethylrhodaminealbumin imaged with Gaussian beam illumination. Automated segmentation was based on simple thresholding. See Figure 6*H* for details.

**Movie 8.** 3D projection of segmented data of the vasculature of a Thy1-GFP-M mouse labelled with tetramethylrhodaminealbumin imaged with Bessel beam illumination. Automated segmentation was based on simple thresholding. See Figure 6*I* for details.

## Discussion

A particular interest in the neuroscience community is to map quantitative data of the whole mouse brain onto common atlases, a task for which LSM is particularly well suited once the mouse brain has been appropriately rendered transparent. Due to the high frame rates obtainable with LSM, whole mouse brain datasets now routinely comprise several terabytes, a size which demands automated tools (Frasconi et al., 2014; Peng et al., 2017) to count, trace, or segment features of interest either to obtain cyto-architectonic information over a mouse brain-wide scale or in the emerging field of digital 3D histology (Torres et al., 2014) to provide automated interpretation of images used for quantitative diagnosis (Bucur et al., 2015). Isolating fluorescent features of interest in a heterogeneous background places higher computational demands on the algorithms used, often with concurrent increase in computation time and complexity of the parameters to be tuned. A recent study showed that very simple algorithms like global thresholding or high pass filtering require uniform background intensity and fail to segment simple fluorescent forms like cell nuclei when a striated background simulating muscle fibers is added to the image (Chitalia et al., 2016). As the complexity of the fluorescent feature increases, so do the demands on the algorithms tasked to isolate them. For instance, neuron tracing is a fundamental tool to understand neuronal morphology and function, however, the accurate segmentation of neurons is to date a challenging task due to their often complex arborization and the varying quality of microscope images (Acciai et al., 2016).

Another recent study compared automated segmentation of a simple synthetic interrupted tube with progressively added salt and pepper noise by a range of published algorithm and their failure to accurately trace this simulated neurite at noise levels of five percentage (Liu et al., 2016). In Figure 5, we show how a threshold of 5% leads to more than a third of the image encompassing half a mouse brain to be affected by a striated background, putting at risk hours of microscope acquisition time, days of data post-processing and weeks of sample preparation. In Figure 6, we have shown that, due to shadowing, image content, especially of finer vessels illuminated with a Gaussian beam, can drop to little above 40% compared to data acquired with Bessel beam illumination, again jeopardizing entire terabyte-sized datasets. By contrast, the quality of the datasets obtained with Bessel beam illumination allowed, for example, the automated segmentation of blood vessels by simple thresholding in areas completely obscured by shadows using Gaussian illumination.

The self-regenerating abilities of Bessel beams are provided by the optical power in the concentric rings, which can reconstruct the original beam profile if the central lobe encounters any obstruction. This property makes Bessel beams attractive to counteract the various causes of striping artefacts as presented in Figure 4. Perfecting the degree of optical clearing in itself is not a universal solution to improved image quality since other mechanism are also at play such as absorption by bright structures of the brain itself or refraction by bubbles. However, the self-healing properties of Bessel beams have to be paid with a reduction in axial sectioning. In our setup, Bessel beam illumination suffered from poorer axial sectioning (Fig. 3*E*) caused by the excitation of fluorescence by the optical power stored in the concentric rings. Optical sectioning could have been improved by employing segmented Bessel beams (Fahrbach et al., 2013b), or by using non-linear excitation strategies (Planchon et al., 2011). These strategies will however introduce further complexity to the optical system and increase the overall cost of the apparatus. In any case, our standard Bessel-beam light-sheet microscope is capable of producing much more uniform images at the cost of an axial resolution only slightly worse than its Gaussian counterpart.

In conclusion, we have illustrated how streaky artefacts introduced by Gaussian illumination can adversely affect and even nullify data extracted from LSM images. We compared the performance of Gaussian and Bessel beam illumination in structural studies, covering brain-wide morphology of neuronal and vascular networks in optically cleared mouse brains. We have found that over a third of the tested volume was adversely affected by illumination inhomogeneity and that, in the worst case, microscopic features of interest are irrecoverably lost. We have shown how the use of Bessel beams can provide an optical solution to correct for these artifacts on the microscope system side and allow for high-fidelity imaging in LSM. The results presented here redefine the quality standard for quantitative measurements in LSM with a single neuron sensitivity that opens up a new class of experimental studies.

## Synthesis

Reviewing Editor: Menno Witter, Norwegian University of Science and Technology Decisions are customarily a result of the Reviewing Editor and the peer reviewers coming together and discussing their recommendations until a consensus is reached. When revisions are invited, a fact-based synthesis statement explaining their decision and outlining what is needed to prepare a revision will be listed below. The following reviewer(s) agreed to reveal their identity: Stéphane Pagès, Anne Schueth. Note: If this manuscript was transferred from JNeurosci and a decision was made to accept the manuscript without peer review, a brief statement to this effect will instead be what is listed below.

In this paper, the authors show how using Bessel beams as illumination source in a Light Sheet microscope (LSM) reduces streaking artefacts in the images. As they state themselves, using Bessel beams in combination with Light Sheet Imaging is not new. The originality of this paper lies in the observation they have made that this imaging arrangement leads to a significant reduction in streaking artefacts usually present on images recorded with a LSM, thus restoring the quality of the image. This is especially interesting and important for large-sized light-sheet imaging data files, as high-fidelity imaging has the potential to relax computational demands on the algorithm used directly.

Major points :

1. Both Gaussian Beams and Bessel beams are mentioned, but not explained very well. The community of readers of eNeuro might not have enough (physics) background knowledge.

2. Authors should discuss in the main text the axial and in plane optical resolution of the microscope with Gaussian and Bessel illumination. Data are presented in Fig. 1 but not discussed. They should explain why the axial resolution with Bessel beam is equivalent and not lower than with Gaussian beam.

3. The amount of shadows present in LSM images depends mainly on the quality of the optical clearing. The brain presented on Figure 2a) may not be representative of typical cleared tissue. The authors just imaged one brain for neuronal imaging and one brain for vasculature imaging. We recommend to measure the variability on the presence of such streaking artifacts in between brain, and provide some numerical data/statistics on the presence of these shadows in imaging.

4. To reinforce the impact of the paper, authors should provide images of a section of the whole brain imaged with Gaussian and Bessel beams. This is absent from the present version of the paper.

5. Comparing Fig 2 d) and e) on one hand and Fig.3 b) and c) on the other hand, it appears that neuronal structures (neurons and dendrites, Fig2) and blood vessels (Fig. 3) are present on images recorded with the Bessel beams but not on those recorded with Gaussian beams. As the axial resolution as presented in Fig 1 does not change significantly between Bessel and Gaussian illumination, where do these structures come from? Could they come from Bessel side lobes excitation (Extended Depth of Field like microscopy)?

6. Authors should explain why they chose a threshold of 5 % in the quantification of streaking artefacts. It would be interesting for readers to know the sensitivity of affected area on this threshold.

7. Overall, the paper remains quite descriptive. Could authors provide an explanation of the observed phenomenon? They should explain why using Bessel beam for illumination helps in reducing the shadows. Could they optimize it?

8. Authors write: “It is apparent that in all images obtained using Gaussian illumination image homogeneity is strongly affected and dark shadows obscure microscopic anatomical features of interest. Notably those same identical features remain clearly visible when using Bessel beam illumination. » Then, it would be interesting to show such examples.

9. On Fig. 2 h) Authors should show an example image of an affected area as defined by their analysis.

10. On Fig. 3a), inside the white square, there is no shadow visible while there is a large dark band in the inset Fig3 b). Authors should provide an explanation for this discrepancy.

11. Resolution of presented images is too low for the reader to figure out the quality of such imaging.

Minor comments

1. LSM as abbreviation of light-sheet microscopy should be used throughout the entire manuscript

2. The authors talk about ‘clarified’ samples, but not only ‘CLARITY’ references are given, so it should rather be ‘optically cleared’ samples, which is more broad.

3. Rephrase the last sentence of the significance statement; in its present form is does not relate to the previous sentences. It is more written as a concluding statement, but that is not what the significance statement is about.

4. Rephrase the sentence in the abstract: ‘We found that over a third of the tested brain volume’ into a third of the imaged volume of the brain

5. Some typographic errors need to be corrected throughout the paper

6. Introduction:

a. you mention ‘scattering or absorbing obstacles’. Give possible examples please

b. At the end of 2nd paragraph the authors could describe what “could be achieved”, with giving examples, such as detecting cell bodies, performing cell counts, it allows.....What is actually possible with applying your method and what is the (practical) benefit?

c. ...“which only halves the problem”, does not sound very scientific and what do the authors mean with this phrase?

7. Methods:

a. In the methods it is clear that n=1 mouse was used for the perfusion procedure. The authors should add this in the method section (n=1). Later in “Whole brain clearing procedure” they describe ‘fixed mouse brains’ were used. But again no n-number is given.

b. It is described that after TDE clearing the brains were ready for imaging. In what solution were they imaged?

c. Is the title “microscope characterisation” correct/clear? Reading this I would have expected a description of the microscope (hardware etc). Maybe the authors could name this section PSF measurement for...

d. No supplier for the beads is given, or are they in house developed?

e. In the image stitching section the correct Fiji reference is missing (Schindelin et al, 2012).

f. Working distance of XLPLN10XSVMP may be wrong. It is probably 8 mm and not 12 mm/are the characteristics of the objectives correct (WD 12mm, RI 1,33~1,52).

g. Check Starna cuvette ref 3/Q/15/TW ... is TW really in the reference?

h. The softwares mentioned are Terastitcher and Zetastitcher, the latter having been developed by the authors in house. The authors should include a comparison between them.

8. Results:

a. in the text the authors describe that for vasculature imaging, an excitation of λ=561nm was used. Which fluorophore/What was excited?

b. Is is correct to describe TDE/PBS as mounting medium?

9. Figures:

a. Fig. 1: The legend should be a bit more detailed. As of now the reader needs extra information (e.g. in the main manuscript).

b. Fig. 2: the authors mention the FOS-trap-mouse/abbreviation without any additional explanation. This is not clear, standing alone.
